# Three-year cardiovascular risk prediction among people who use cocaine or methamphetamine

**DOI:** 10.1016/j.dadr.2026.100459

**Published:** 2026-06-22

**Authors:** Rebecca Arden Harris, Fengge Wang, Warren B. Bilker, Renae Judy, Michael G. Levin, Scott M. Damrauer, Sean Hennessy

**Affiliations:** a Department of Family Medicine and Community Health, Perelman School of Medicine, University of Pennsylvania, Philadelphia, PA, USA; b Penn Cardiovascular Institute, University of Pennsylvania, Philadelphia, PA, USA; c Leonard Davis Institute of Health Economics, University of Pennsylvania, Philadelphia, PA, USA; d Department of Biostatistics and Epidemiology, University of Pennsylvania, Philadelphia, PA, USA; e Department of Surgery, Perelman School of Medicine, University of Pennsylvania, Philadelphia, PA, USA; f Division of Cardiovascular Medicine, Perelman School of Medicine, University of Pennsylvania, Philadelphia, PA, USA; g Michael J. Crescenz VA Medical Center, Philadelphia, PA, USA; h Center for Real-World Effectiveness and Safety of Therapeutics, Department of Biostatistics, Epidemiology and Informatics, Perelman School of Medicine, University of Pennsylvania,Philadelphia, PA, USA

**Keywords:** Risk assessment, Cocaine, Methamphetamine, Stimulants, Stimulant use disorder, Cardiovascular disease

## Abstract

**Background:**

Cardiovascular disease (CVD) risk prediction tools have not been developed or validated for adults who use nonmedical stimulants (cocaine or methamphetamine), despite substantially elevated event rates and pathophysiologically distinct risk profiles in this population. Accurate estimation of absolute risk is needed to support individualized clinical decision-making.

**Methods:**

We developed a 3-year CVD risk prediction model using a cohort of 6940 adults aged 18–79 with documented stimulant use, identified from electronic health record (EHR) data of a large academic hospital system spanning 2016–2024. Type of stimulants used was a candidate predictor, alongside demographic characteristics, cigarette smoking status, systolic blood pressure, and baseline cardiovascular medications. Variable selection used least absolute shrinkage and selection operator (LASSO) with 10-fold cross-validation, followed by unpenalized logistic regression on selected variables. Model performance was assessed using discrimination and calibration metrics; bootstrap internal validation estimated and corrected for optimism in apparent performance.

**Results:**

LASSO selected 8 predictors: age, sex, Black race, Hispanic ethnicity, current cigarette smoking, cocaine-only use, any cardiovascular medication use, and systolic blood pressure. Calibration was excellent. Observed and predicted 3-year event rates were 29.6% and 30.1%, respectively (observed/expected [O/E] ratio 0.985, calibration slope 1.000, integrated calibration index [ICI] 0.008). Performance was maintained after optimism correction: O/E ratio 0.988, calibration slope 0.992, ICI 0.010. The apparent C-statistic was 0.730 (95% CI 0.717–0.743); the optimism-corrected C-statistic was 0.728. For illustration, a 45-year-old Black adult with cocaine use, current cigarette smoking, and systolic blood pressure of 130 mmHg has an estimated 3-year CVD risk of approximately 27.7%; the same profile with methamphetamine use yields approximately 22.1%.

**Conclusions:**

A CVD risk prediction model tailored to individuals with stimulant use accurately estimated absolute 3-year CVD risk, with excellent calibration and clinically meaningful risk stratification. These findings establish proof of concept for a stimulant-specific CVD risk prediction tool. External validation and implementation studies are needed before clinical deployment.

## Graphical Abstract

**Figure:**
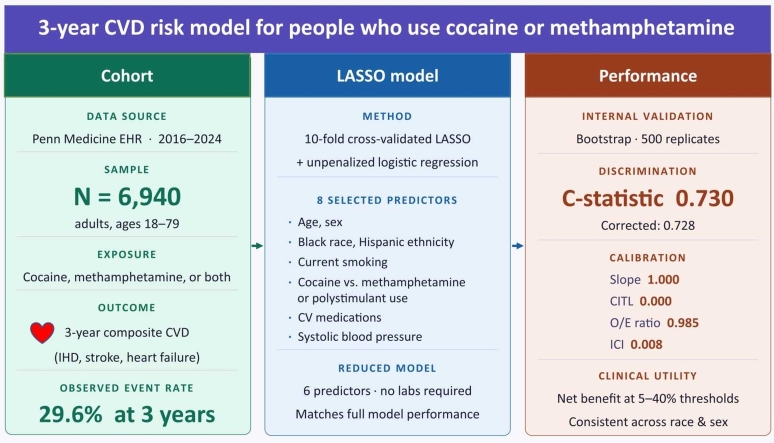

## 1 Introduction

The health burden of nonmedical stimulant use, specifically cocaine and methamphetamine, has reached historic levels in the United States (Tanz et al., 2025). In 2024, an estimated 4.3 million individuals aged 12 and older reported past-year cocaine use, and 2.4 million reported methamphetamine use; of these, approximately 2.6 million met diagnostic criteria for stimulant use disorder (Substance Abuse and Mental Health Services Administration SAMHSA, 2025; authors' calculation). Deaths involving stimulants without opioid co-involvement increased by 251% nationally between 2014 and 2023, with many of these deaths stemming from cardiovascular complications including myocardial infarction, arrhythmias, cardiomyopathy, and stroke (Harris et al., 2025b).

Both cocaine and methamphetamine cause multifaceted cardiovascular injury through acute and chronic mechanisms. Acutely, catecholamine excess produces tachycardia, hypertension, and coronary vasoconstriction, precipitating acute coronary syndromes even in angiographically normal vessels; cocaine additionally promotes thrombosis through platelet activation and impaired fibrinolysis (Havakuk et al., 2017, Kim and Park, 2019, Pergolizzi et al., 2021). With chronic exposure, both drugs accelerate atherosclerosis and exert direct myocardial toxicity through oxidative stress, endothelial injury, and adverse ventricular remodeling (Sandfort et al., 2017, Lai et al., 2023, Kevil et al., 2019). Cocaine use is robustly linked to cardiomyopathy, while methamphetamine-associated cardiomyopathy has emerged as a distinct clinical entity characterized by dilated cardiomyopathy with reduced ejection fraction and frequent pulmonary arterial hypertension (Arenas et al., 2020, Sharma et al., 2020, Reddy et al., 2020, Bhatia et al., 2021, Kolaitis et al., 2022, Shetty et al., 2021, Winkelman et al., 2018, Curran et al., 2022). Both agents confer markedly increased risk of stroke through acute hypertension, vasospasm, and thrombotic mechanisms, and are associated with arrhythmias including sudden cardiac death through direct arrhythmogenic effects (Havakuk et al., 2017, Roa et al., 2024, Dominic et al., 2022, Duflou, 2020). Clinically, both are linked to early-onset cardiovascular disease disproportionately affecting individuals under 45 years (O'Keefe et al., 2022, Batra et al., 2022, Tobolski et al., 2022). Despite this well-characterized and escalating burden, no cardiovascular risk prediction tool has been developed or validated specifically for individuals who use cocaine or methamphetamine, nor have existing models been evaluated in this population.

The American Heart Association's Predicting Risk of Cardiovascular Disease EVENTs (PREVENT) equations, introduced in 2023, represent the current standard for 10- and 30-year cardiovascular risk estimation in the general population. PREVENT improved upon earlier models by incorporating kidney function and predicting both atherosclerotic cardiovascular disease and heart failure (Khan et al., 2023, Khan et al., 2024). However, PREVENT was developed in cohorts with few individuals with substance use disorders. Standard prediction models are insufficient for several reasons. Cardiovascular events in people who use stimulants arise not only from accelerated atherosclerosis but also from acute toxic effects and arrhythmic mechanisms not captured by traditional risk factors. People who use stimulants also develop disease at substantially younger ages, and stimulant-specific variables such as type of stimulant and polysubstance patterns are absent from existing models.

This gap has direct consequences for clinical practice. Cardiovascular risk estimation informs shared decision-making about statin initiation, blood pressure targets, and lifestyle interventions. For patients who use stimulants, clinicians currently lack evidence-based tools to quantify cardiovascular risk or to target preventive interventions to those at highest risk. This need is most acute for patients already engaged in care, where a clinician can act on risk information, and where stimulant use is clinically known. A well-calibrated prediction model could serve multiple purposes: identifying which patients face the highest near-term cardiovascular risk and would benefit most from intensive preventive interventions, and providing patients with personalized risk information that contextualizes the cardiovascular consequences of ongoing stimulant use.

We developed and internally validated a 3-year cardiovascular risk prediction model for adults who use cocaine or methamphetamine, using electronic health record (EHR) data from a large academic health system. We focused on a 3-year time horizon for several reasons. First, stimulant-associated cardiovascular pathophysiology operates through acute mechanisms including catecholamine excess and vasospasm that may accelerate event timing relative to traditional risk factors. Second, shorter prediction windows reduce the influence of unmeasured changes in predictors occurring between baseline assessment and outcomes. Third, shorter horizons minimize bias from competing events including death from non-cardiovascular causes and loss to follow-up due to incarceration or care discontinuity, for which we lacked complete data. Our primary aim was to examine proof of concept for a well-calibrated model suitable for clinical decision support, recognizing that calibration (the agreement between predicted and observed risk) is the property most essential for guiding individual patient care (Alba et al., 2017).

## 2 Methods

### 2.1 Study design and setting

The design, conduct, analyses, and reporting of this study adhered to the Transparent Reporting of a Multivariable Prediction Model for Individual Prognosis or Diagnosis (TRIPOD) guidelines (Collins et al., 2015, Moons et al., 2015) and to methodological guidance for the evaluation and internal validation of clinical prediction models (Binuya et al., 2022). Our data curation and analytic procedures were consistent with PREVENT specifications, with adaptations appropriate to our study population and available EHR data. We conducted a retrospective cohort study using EHR data from the University of Pennsylvania Health System (Penn Medicine), which comprises six hospitals and a network of multispecialty outpatient centers serving south-central Pennsylvania, south-central New Jersey, and northern Delaware (Cho et al., 2025 Jul 22, Goldstein et al., 2017, Lee et al., 2020). The study period spanned January 1, 2016, through December 31, 2024. All analyses were restricted to adults with documented cocaine or methamphetamine use; stimulant use defines the study cohort rather than serving as a predictor variable within a general population sample.

#### 2.1.1 Ethical review

This study used de-identified EHR data provided through the Penn Data and Analytics Center (PennDnA). Under University of Pennsylvania Institutional Review Board policy, fully de-identified datasets that contain no direct identifiers or reasonable basis for re-identification do not constitute human subjects research. All data were accessed and analyzed in de-identified form, with no attempt to re-identify patients.

### 2.2 Study Population

Based on electronic health records, we identified adults aged 18–79 years with documented stimulant use at baseline. Stimulant use was defined as cocaine or methamphetamine use identified through the International Classification of Diseases, Tenth Revision (ICD−10) diagnosis codes for cocaine-related disorders (F14.x) or use (T40.5x) or amphetamine-related disorders (F15.x) or use (T43.6x)(Shearer et al., 2021; Rowe et al., 2021; Paulus and Stewart, 2020; Srivastava et al., 2025). The index date was defined as the date of the clinical encounter at which the first ICD code for stimulant use was recorded in the EHR.

We designed the model for a clinically engaged population (adults whose stimulant use is known to their care team), which is both the appropriate scope for a point-of-care risk tool and the population in whom such a tool would realistically be deployed. Patients whose stimulant use is undocumented or who are not engaged in care are outside the intended target population of the model.

From an initial cohort of patients with documented stimulant use, we applied the following exclusion criteria sequentially. We excluded patients outside the age range of 18–79 years. We excluded patients whose only health system contact was the index encounter, as longitudinal risk prediction requires at least one subsequent clinical encounter to ascertain outcome status. We excluded patients for whom a CVD event occurred on or before the index date encounter, as these represent prevalent rather than incident events. To further reduce the likelihood of reverse causation, whereby early cardiovascular symptoms may have prompted the clinical encounter at which stimulant use was first documented, we further excluded patients with a CVD event within 30 days of the index date.

### 2.3 Outcome

The primary outcome was a composite cardiovascular event occurring between 30 days and 3 years (1095 days) after the index date, defined by the earliest qualifying event. The main composite comprises fatal and nonfatal myocardial ischemia, cerebrovascular disease, and heart failure, following the AHA PREVENT framework. We expanded PREVENT's myocardial infarction (MI) codes (ICD−10 I21–I22) to I20–I25 and stroke codes (I61–I63) to I60–I69, following CDC National Cardiovascular Disease Surveillance System indicator definitions (Centers for Disease Control CDC, 2004), to capture the broader ischemic and cerebrovascular presentations (including vasospasm, unstable angina, and hemorrhagic stroke) that characterize stimulant-related cardiovascular injury and are not captured by atherosclerosis-focused code sets; heart failure (I50) was retained unchanged. A secondary composite additionally includes cardiac arrhythmias (ICD−10 I44–I49), given evidence that stimulants have direct arrhythmogenic effects. Supplementary Table S1 compares our ICD−10 codes with those used in the AHA PREVENT equations.

We selected a 3-year prediction horizon for three reasons: (1) stimulant-associated cardiovascular pathophysiology operates through acute mechanisms that may accelerate event timing; (2) shorter prediction windows reduce the influence of unmeasured changes in predictors between baseline assessment and outcomes; and (3) a shorter horizon minimizes bias from competing events, including non-cardiovascular death, incarceration, and care discontinuity, for which complete data were unavailable.

Because 3-year event status could not be ascertained for patients who left the health system without a recorded outcome, the analytic sample was restricted to patients who either experienced a cardiovascular event within the follow-up window or had at least one healthcare encounter after 3 years, suggesting event-free survival. Patients meeting neither criterion were excluded.

### 2.4 Predictors

All predictor values were ascertained from measurements obtained within 30 days of the index date. For variables with multiple measurements in this window, we used the value closest to the index date. Candidate predictors were prioritized for clinical relevance and data completeness in EHR data. Total cholesterol, high density lipoprotein (HDL) cholesterol, estimated glomerular filtration rate (eGFR), and hemoglobin A1c (HbA1c) were not included due to high rates of missing values. These omissions were prespecified to prioritize model stability and transportability to real-world EHR settings where laboratory data availability varies. The final predictor set therefore emphasizes routinely available structured data (demographics, vitals, cigarette smoking, medication use, and stimulant use documentation) while remaining conceptually consistent with PREVENT variables where data permitted. This pragmatic approach yields a model suited for point-of-care implementation in clinical settings with fragmented laboratory data, though it shifts emphasis toward prognostic rather than purely preventive risk estimation.

PREVENT-equivalent variables included age, sex, systolic blood pressure, and current cigarette smoking status. Smoking status was classified as current versus former/never based on structured EHR documentation.

#### 2.4.1 Missing data

Exclusion of lipid panels, HbA1c, and eGFR was justified on both analytic and clinical grounds (Heymans and Twisk, 2022, Sisk et al., 2023). A prediction tool requiring routine lipid and glycemic laboratory values would be difficult to apply consistently in the settings most likely to serve patients with stimulant use disorders (Little and Rubin, 2019, Lee et al., 2021).

The 761 patients excluded for missing systolic blood pressure represent the one required predictor for which imputation might have been considered. A regression of systolic blood pressure on all available covariates in the analytic sample, however, yielded R² = 0.11 (root mean squared error [RMSE] = 18.7 mmHg), indicating that imputed values would carry uncertainty of approximately 37 mmHg at the 95% level, too imprecise to meaningfully improve model performance relative to complete-case analysis.

Restricting derivation to participants with complete data on required predictors is consistent with the approach used in developing the PREVENT equations (Khan et al., 2024). Unlike PREVENT, we excluded lipid panels and hemoglobin A1c because their high missingness in this cohort would have substantially reduced the analytic sample and risked selection bias by conditioning inclusion on laboratory availability. PREVENT retained lipid panels as required inputs but did not report missingness rates across its 46 derivation and validation cohorts (Khan et al., 2024; see Table 1 in Hong et al., 2026, in which missingness fields for the PREVENT cohorts are listed as not available). The scale of this problem is suggested by the optional predictor hemoglobin A1c, which was missing in 100% of several major research cohorts and in 35–78% of the large clinical database cohorts (Khan et al., 2024, Supplemental Appendix 1.4, Table S19), patterns that strongly suggest missingness for required predictors such as lipids was likely considerable as well. In a large EHR validation cohort at Duke University Health System, lipid data were missing in approximately 52% of patients prior to applying complete-case restrictions (Hong et al., 2026).

**Table 1:** Baseline characteristics of the study population.

**Characteristic**	**N = 6940**
**Demographics**	
Age, mean (SD), years	45.4 (12.9)
Female sex, n (%)	2844 (41.0)
Race/Ethnicity, n (%)	
Asian	38 (0.6)
Black	4109 (59.2)
White	2443 (35.2)
More than one race	83 (1.2)
Other/Unknown race	267 (3.8)
Hispanic	295 (4.3)
**Cardiovascular Risk Factors**	
Current cigarette smoking, n (%)	3922 (56.5)
Systolic blood pressure, mean (SD), mmHg	129.3 (19.7)
**Cardiovascular Medications**^**a**^	
Lipid lowering medication	443 (6.4)
Anti-hypertensive medication:	
Angiotensin-converting enzyme inhibitor, n (%)	333 (4.8)
Angiotensin receptor blocker, n (%)	160 (2.3)
Beta-blocker, n (%)	252 (3.6)
Calcium channel blocker, n (%)	616 (8.9)
Any cardiovascular medication, n (%)	1169 (16.8)
**Stimulant Use**	
Cocaine use w/o methamphetamine, n (%)	5604 (80.8)
Methamphetamine use w/o cocaine, n (%)	1075 (15.5)
Cocaine and methamphetamine use, n (%)	261 (3.8)
**3-Year Cardiovascular Outcomes**	
Arrhythmia, n (%)	2243 (32.3)
Cerebrovascular disease, n (%)	1075 (15.5)
Heart failure, n (%)	832 (12.0)
Ischemic heart disease, n (%)	1142 (16.5)
Any cardiovascular outcome w/o arrhythmia⁎, n (%)	2056 (29.6)
Any cardiovascular outcome w/ arrhythmia⁎, n (%)	3179 (45.8)

Abbreviation: SD, standard deviation. ^⁎^Our main composite outcome comprised ischemic heart disease, cerebrovascular disease, and heart failure (consistent with AHA PREVENT). A composite including arrhythmia was examined separately given its direct association with stimulant use. Patients may have experienced more than one component event.

### 2.5 Statistical analysis: model development

We used a two-stage penalized regression approach to estimate 3-year cardiovascular risk. In the first stage, least absolute shrinkage and selection operator (LASSO) with 10-fold cross-validation was used for variable selection. LASSO simultaneously selects predictors and shrinks coefficients toward zero, reducing overfitting risk when the number of candidate predictors is large relative to sample size. The outcome was modeled as binary (cardiovascular event within 3 years vs. no event). All continuous predictors were standardized to mean 0 and standard deviation 1 prior to model fitting to ensure equal penalization across predictors of different scales. The optimal regularization parameter (λ) was selected by minimizing binomial deviance under 10-fold cross-validation (λ-min criterion).

In the second stage, the predictors retained by LASSO were entered into a standard unpenalized logistic regression model, yielding interpretable odds ratios with confidence intervals unaffected by penalization-induced shrinkage (Pavlou et al., 2016).

#### 2.5.1 Internal Validation

To quantify optimism in apparent model performance, we conducted bootstrap internal validation using Harrell's optimism-correction method with 500 replicates. In each replicate, a bootstrap sample was drawn with replacement from the full analytic cohort, and the complete two-stage modeling procedure (LASSO variable selection followed by unpenalized logistic regression) was repeated on the bootstrap sample, with continuous predictors re-standardized using the bootstrap sample's own mean and standard deviation. Apparent performance was assessed on the bootstrap sample, and the fitted model was then applied to the original dataset to estimate test performance. Per-replicate optimism was defined as the difference between apparent and test performance; optimism-corrected point estimates were obtained by subtracting mean optimism (averaged across 500 replicates) from the apparent performance on the full analytic cohort.

Confidence intervals were derived using different methods depending on the metric. For the C-statistic (apparent), we used the asymptotic normal approximation from the receiver operating characteristic (ROC) curve. For calibration-in-the-large and calibration slope (apparent), confidence intervals were derived analytically from the standard errors of the intercept and slope terms in the calibration regression. For the Brier score (apparent), we used a delta-method standard error (standard deviation of individual squared errors divided by the square root of N). For the observed-to-expected ratio and integrated calibration index, no closed-form standard error is available, so confidence intervals were derived from bootstrap percentiles. For all optimism-corrected estimates, confidence intervals were derived from the 2.5th and 97.5th percentiles of the replicate-level corrected distribution across 500 bootstrap replicates.

#### 2.5.2 Performance metrics

Calibration was assessed as the primary performance metric, given our objective of producing a model suitable for individualized clinical decision support. We evaluated calibration using:

• Calibration plots displaying observed event proportions against mean predicted probabilities across deciles of predicted risk (perfect calibration produces points along the 45° diagonal) (Bonnett et al., 2019).
• Calibration slope, quantifying agreement between predicted and observed risk across the prediction spectrum (slope = 1 indicates perfect calibration; <1 suggests overfitting).
• Calibration-in-the-large (CITL), assessing whether mean predicted risk equals mean observed risk (CITL = 0 indicates no systematic bias).
• Integrated calibration index (ICI), the weighted average absolute difference between smoothed observed and predicted probabilities.
• Observed/Expected (O/E) ratio, providing global calibration assessment (1.0 = perfect; >1 = overprediction; <1 = underprediction).
• Brier score, measuring mean squared difference between predicted probabilities and observed outcomes (lower values indicate better accuracy).

Discrimination was assessed using the C-statistic (area under the receiver operating characteristic curve). Because the upper bound on the C-statistic is inversely related to the length of the prediction horizon – a relationship observed empirically across risk prediction models evaluated at multiple time points (Rahimi et al., 2014) – C-statistics from our 3-year model are not directly comparable to those from conventional 10- or 30-year models (Austin et al., 2017).

Performance metrics were calculated at both the apparent and optimism-corrected stage.

#### 2.5.3 Subgroup performance analysis

To evaluate model fairness and assess whether performance was consistent across clinically relevant subgroups, we prespecified examination of discrimination and calibration separately in subgroups defined by race (Black, White) and sex (female, male). Subgroups were selected based on clinical relevance and adequate sample size for meaningful performance assessment. Performance metrics (C-statistic, observed/expected [O/E] ratio, CITL, calibration slope, ICI, and Brier score) were calculated using apparent model predictions from the full 8-predictor model applied to each subgroup. Bootstrap optimism correction was not applied at the subgroup level, as the primary purpose of this analysis was descriptive assessment of subgroup-specific calibration and discrimination rather than bias-corrected estimation.

We additionally examined model performance by stimulant type (cocaine-only, methamphetamine-only, and poly-stimulant use), given that stimulant type is anticipated to be both a key predictor in the model and a potential source of differential performance: the pathophysiological mechanisms and event timelines differ between cocaine and methamphetamine, and the model's ability to provide well-calibrated risk estimates within each stimulant subgroup is directly relevant to its clinical applicability across the range of patients likely to be encountered in practice.

#### 2.5.4 Sample Size Considerations

We did not conduct a formal sample size calculation a priori, as the study used all available data meeting inclusion criteria (Ogundimu et al., 2016). However, we assessed the adequacy of sample size using the events-per-variable (EPV) framework (Austin and Steyerberg, 2017). Contemporary guidance suggests that prediction models require approximately 10–20 events per candidate predictor to avoid overfitting, though LASSO regularization permits stable estimation with somewhat lower EPV. We report the number of outcome events and the ratio of events to candidate predictors (prior to LASSO selection) to contextualize model stability (Dhiman et al., 2023, Riley et al., 2019, Riley et al., 2020).

#### 2.5.5 Reduced model development

We prespecified development of a clinically simplified model following internal validation of the full model, with the goal of producing a parsimonious tool suitable for point-of-care use without laboratory testing (Steyerberg et al., 2010, Steyerberg, 2019). Criteria for predictor exclusion were established prior to analysis and included: small subgroup size precluding stable coefficient estimation, no meaningful association with the outcome, and minimal impact on model discrimination when removed. The specific predictors meeting these criteria were determined after model fitting. The reduced model was refit on the full analytic cohort using standard unpenalized logistic regression. Performance was assessed using the same metrics and bootstrap optimism correction procedure applied to the full model. To facilitate point-of-care implementation, model coefficients were re-estimated on unstandardized predictors and expressed as a closed-form logistic equation suitable for use in a spreadsheet-based calculator.

#### 2.5.6 Threshold-dependent classification metrics

To characterize model utility across a range of clinical decision thresholds, we prespecified calculation of sensitivity, specificity, positive predictive value (PPV), negative predictive value (NPV), and the proportion of patients classified as higher- and lower-risk at predicted probability thresholds of 20%, 30%, and 40%. These thresholds were selected to span the range of clinical utility identified in the decision curve analysis and to bracket the observed 3-year event rate. These metrics are reported for the reduced predictor model to support point-of-care implementation.

#### 2.5.7 Software

Statistical analyses were conducted in Stata 18.5 (College Station, Texas).

## 3 Results

### 3.1 Study population

**Fig. 1:**
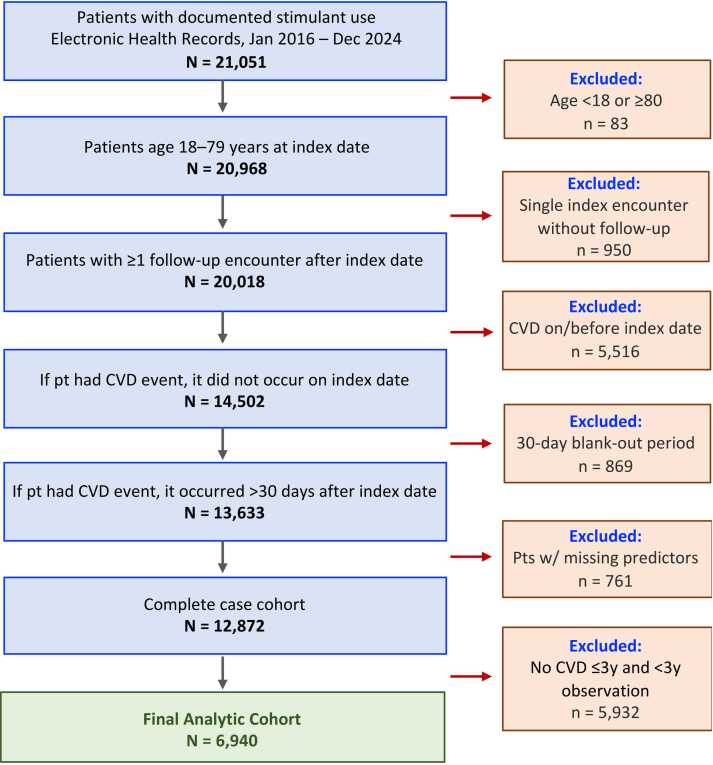
Cohort selection flow diagram.

From an initial cohort of 21,051 patients with documented stimulant use, we applied our sequential exclusion criteria, which yielded a final analytic sample of 6940 patients. Fig. 1 diagrams the cohort selection flow and Table 1 presents the baseline characteristics. The mean age was 45.4 years (SD 12.9); 41.0% were women. The majority of patients identified as Black (59.2%), with 35.2% identifying as White. Current cigarette smoking was documented in 56.5% of patients. Cocaine without methamphetamine was the predominant stimulant type (80.8%), followed by methamphetamine without cocaine (15.5%) and concurrent use of both (3.8%). Most patients had at least one F14 or F15 disorder code (99.1%); the remaining patients (0.9%) were identified through T40.5x or T43.6x poisoning codes. Mean systolic blood pressure was 129.3 mmHg (SD 19.7). Overall, 16.8% of patients had at least one cardiovascular medication prescription; calcium channel blockers were the most commonly prescribed antihypertensive (8.9%), followed by angiotensin-converting enzyme (ACE) inhibitors (4.8%), beta-blockers (3.6%), and angiotensin receptor blockers (ARBs) (2.3%).

**Fig. 2:**
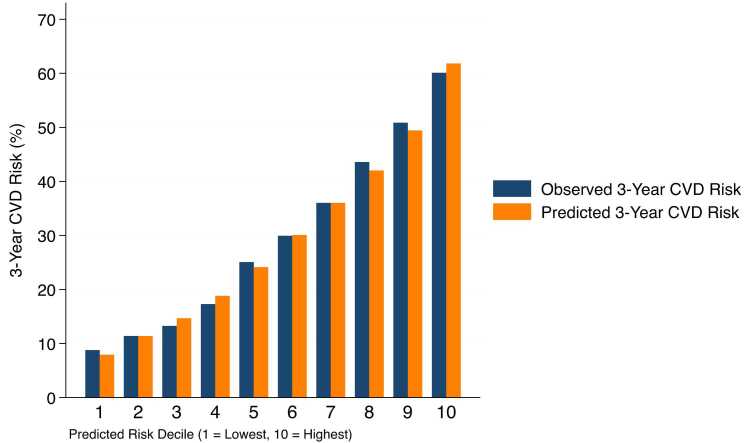
Calibration plot: Observed vs. predicted 3-Year cardiovascular risk by decile of predicted risk. Bars represent observed (blue) and predicted (orange) 3-year cardiovascular event rates within each decile of predicted risk. Decile 1 represents the lowest-risk patients; decile 10 represents the highest-risk patients. Close alignment between observed and predicted values across all deciles indicates good model calibration.

### 3.2 Outcomes

During the 3-year follow-up period, 29.6% of patients experienced the primary composite outcome of ischemic heart disease, cerebrovascular disease, or heart failure; this rose to 45.8% when arrhythmia was included. Ischemic heart disease was the most common component (16.5%), followed by cerebrovascular disease and heart failure (15.5% and 12.0%, respectively). Arrhythmia was the most frequent individual event overall, occurring in 32.3% of patients.

### 3.3 Model development

LASSO logistic regression with 10-fold cross-validation selected eight predictors at the optimal λ of 0.0031: age, sex, Black race, Hispanic ethnicity, current cigarette smoking, cocaine use, any cardiovascular medication use, and systolic blood pressure. Post-LASSO logistic regression odds ratios are presented in Table 2.

**Table 2:** Multivariable logistic regression model for 3-Year cardiovascular risk.

**Variable**	**Odds Ratio**	**95% CI**	**P-value**
Age (per SD increase)	2.01	1.88–2.15	< 0.01
Female	1.07	0.96–1.20	0.23
Race: Black (vs. other)	1.11	0.97–1.27	0.13
Hispanic (vs. other)	0.75	0.54–1.05	0.09
Current cigarette smoking (vs. other)	1.16	1.03–1.30	0.01
Cocaine only (vs. methamphetamine or both)	1.36	1.14–1.62	< 0.01
Systolic blood pressure (per SD increase)	1.13	1.07–1.19	< 0.01
Any cardiovascular medication	1.82	1.59–2.10	< 0.01

Abbreviations: CI, confidence interval; SD, standard deviation. Variables selected by least absolute shrinkage and selection operator (LASSO). Final coefficients estimated by post-selection unpenalized logistic regression. Age and systolic blood pressure were standardized (mean 0, SD 1) prior to model fitting to place them on a common scale for penalization and to aid in interpretation; odds ratios represent the change in odds per 1 standard deviation in each of these variables.

Age was independently associated with increased risk (OR 2.01, 95% CI 1.88–2.15), as were current cigarette smoking (OR 1.16, 95% CI 1.03–1.29) and cocaine use relative to methamphetamine or polystimulant use (OR 1.36, 95% CI 1.14–1.62). Higher systolic blood pressure was associated with modestly increased risk (OR 1.13, 95% CI 1.07–1.19). Any cardiovascular medication use was associated with increased risk (OR 1.82, 95% CI 1.59–2.10), a finding that likely reflects confounding by indication – marking patients with more severe underlying disease – rather than a causal treatment effect. The ratio of outcome events to selected predictors exceeded 200:1, well above the recommended minimum threshold, supporting model stability (Efthimiou et al., 2024).

### 3.4 Model performance

Performance metrics are summarized in Table 3. The LASSO-selected model demonstrated good discrimination, with an apparent C-statistic of 0.730 (95% CI 0.717–0.743). Bootstrap internal validation (500 replicates) confirmed minimal overfitting, with over-optimism in the C-statistic of 0.002, yielding an optimism-corrected C-statistic of 0.728 (95% CI 0.713–0.741). Calibration was excellent. The Integrated Calibration Index was 0.008 (optimism-corrected: 0.010), indicating that mean absolute deviation between predicted and observed probabilities was less than 1 percentage point across the range of predicted risk. The Brier score was 0.182 (optimism-corrected: 0.182), with a scaled Brier of 0.129, reflecting meaningful improvement over a null model predicting the marginal event rate for all patients.

**Table 3:** Performance of the 3-Year CVD risk prediction model.

**Metric**	**Apparent**	**Optimism-Corrected**
C-statistic (95% CI)	0.730 (0.717–0.743)	0.728 (0.713–0.741)
O/E ratio (95% CI)	0.985 (0.948–1.022)	0.988 (0.952–1.022)
Calibration-in-the-large (95% CI)	0.000 (−0.056–0.056)	0.003 (−0.056–0.060)
Calibration slope (95% CI)	1.000 (0.930–1.070)	0.992 (0.919–1.063)
Integrated Calibration Index (95% CI)	0.008 (0.002–0.018)	0.010 (0.001–0.018)
Brier score (95% CI)	0.182 (0.177–0.186)	0.182 (0.177–0.187)

Abbreviations: CI, confidence interval; O/E, observed/expected. Apparent performance is estimated on the full analytic cohort. Optimism-corrected estimates are derived using Harrell's bootstrap method (500 replicates), in which the complete two-stage modeling procedure was repeated in each replicate. Calibration-in-the-large represents the intercept from a logistic regression of observed outcomes on the log-odds of predicted probability, with the slope constrained to 1; a value of 0 indicates perfect mean calibration. Confidence intervals for the C-statistic (apparent) are asymptotic normal; for calibration-in-the-large and calibration slope (apparent), analytic; for the Brier score (apparent), delta-method; for the O/E ratio and integrated calibration index, bootstrap percentile; and for all optimism-corrected estimates, bootstrap percentile.

### 3.5 Decision curve analysis

**Fig. 3:**
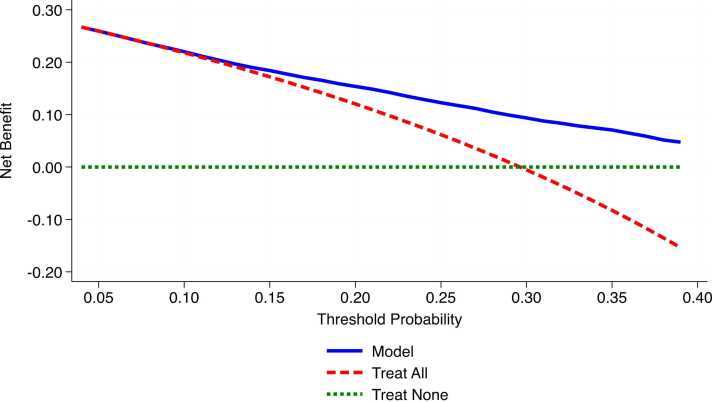
Decision Curve of 3-Year cardiovascular risk. The x-axis represents the minimum predicted 3-year cardiovascular risk at which a clinician would decide to intervene. For example, a threshold of 0.15 means "treat patients whose predicted risk is at least 15%." At low thresholds, the bar for treatment is so permissive that nearly everyone qualifies, approaching the treat-all strategy; at higher thresholds, treatment is concentrated in higher-risk patients only. The y-axis represents net benefit, which weighs the gains from correctly treating high-risk patients against the harms of unnecessarily treating low-risk ones. In this population, approximately 30% develop cardiovascular disease over 3 years. Imagine screening 100 such patients: if all 100 are treated, 70 people are exposed to unnecessary intervention, and the harms outweigh the gains, which is why the treat-all line (dashed red) drops below zero beyond a threshold of roughly 30%. The model (solid blue line) avoids this by identifying which patients are likely to be in that high-risk group, so clinicians can concentrate treatment where it is most likely to help.

Decision curve analysis confirmed the model's clinical utility across a broad range of decision thresholds. Net benefit, a measure that weighs the benefit of correctly identifying high-risk patients (true positives who receive a helpful intervention) against the harm of unnecessarily treating low-risk patients (false positives who bear treatment risks without benefit), was used to compare strategies (Vickers and Elkin, 2006, Vickers et al., 2016). Fig. 3 shows the decision curve of the 3-year risk model. The model (solid blue line) provided greater net benefit than either the treat-all strategy (dashed red line) or the treat-none strategy (dotted green line) at all thresholds between 5% and approximately 38% (Fig. 3). The treat-all strategy yielded positive net benefit only at thresholds below roughly 15%, corresponding to the observed event prevalence, and became increasingly harmful at higher thresholds as the cost of unnecessary treatment outweighed the benefit of event prevention. These findings indicate that the model provides greater net benefit than uniform intervention strategies across the full range of clinically plausible risk thresholds, supporting its potential use as a decision-support tool.

Threshold-dependent performance metrics at predicted probability thresholds of 20%, 30%, and 40%, which span the range of clinical utility identified in the decision curve analysis, are presented in Supplemental Table S2.

### 3.6 Reduced model for point-of-care implementation

Following internal validation of the full model, we conducted a secondary analysis to evaluate whether a clinically simplified model could achieve comparable predictive performance. Two predictors were excluded from the full LASSO-selected model: Hispanic ethnicity, which showed no meaningful association with the outcome and did not contribute to model discrimination, and sex due to its weak effect and minimal impact on model performance when removed. The reduced model retained six predictors (age, Black race, current cigarette smoking, cocaine-only stimulant use, any cardiovascular medication use, and systolic blood pressure) and was refit on the full analytic cohort without penalization.

Performance of the reduced model was essentially identical to the full model across all metrics. The apparent C-statistic was 0.731 (95% CI 0.718–0.743), with an optimism-corrected estimate of 0.729 (95% CI 0.717–0.742). Calibration was excellent: CITL was 0.000 (95% CI −0.055–0.055), calibration slope 1.000 (95% CI 0.931–1.069), and ICI 0.007 (optimism-corrected: 0.008). The Brier score was 0.180 (optimism-corrected: 0.180). These findings indicate that the simplified model sacrifices no meaningful predictive performance while improving clinical implementability.

The predicted 3-year probability of a cardiovascular event is given by:

$$P=\frac{1}{1+e^{-\eta }}$$

where:

*η* = −4.751 + 0.054(Age) + 0.132(Black race) + 0.150(Current cigarette smoker) + 0.301(Cocaine only use) + 0.599(Any CV medication) + 0.006(Systolic BP)

Binary predictors are coded 1 if present and 0 otherwise. Age is entered in years and systolic blood pressure in mmHg. These coefficients are derived from a model fit on unstandardized predictors and are distinct from the standardized coefficients used in the primary analysis. Table S2 presents the diagnostic accuracy metrics (sensitivity, specificity, positive predictive value, and negative predictive value) for the reduced model.

#### 3.6.1 CVD calculator for stimulant use

**Fig. 4:**
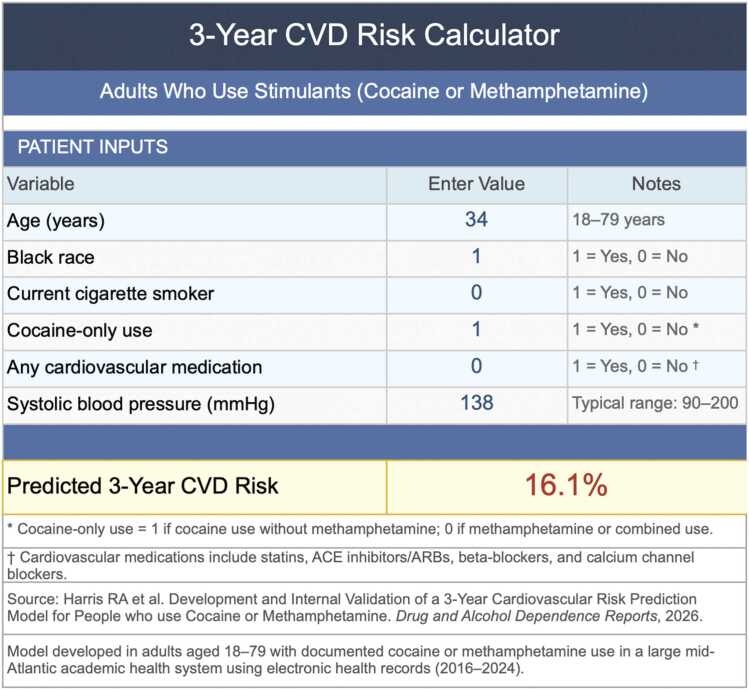
Excel-based 3-year CVD risk calculator for adults who use stimulants.

To facilitate point-of-care application of the reduced model, we developed a Microsoft Excel-based risk calculator (Fig. 4). Clinicians enter six variables (age, Black race, current cigarette smoking status, cocaine-only use, any cardiovascular medication use, and systolic blood pressure) and the tool returns a predicted 3-year CVD risk. No laboratory values are required, reflecting the model's intentional design for settings where lipid panels and glycemic testing are not consistently available. The calculator is currently specific to the population in which the model was derived: adults with documented cocaine or methamphetamine use receiving care at a large academic health system in the mid-Atlantic region. It is included here to illustrate how the model could be used at the point-of-care.

#### 3.6.2 Illustrative examples

Table 4 presents estimated 3-year CVD risk for six hypothetical profiles to further illustrate the model's output across a range of clinically meaningful predictor combinations. These estimates are intended only as an interpretive aid.

**Table 4:** Illustrative 3-year CVD risk estimates for six hypothetical profiles of adults with cocaine or methamphetamine use.

**Profile**	**Age**	**Black** **Race**	**Cigarette** **Smoking**	**Cocaine** **Only**	**CV** **Meds**	**SBP** **(mmHg)**	**3-yr CVD risk**
Young, methamphetamine use, no other risk factors	20	No	No	No	No	115	4.8%
Young, cocaine only, Black	30	Yes	No	Yes	No	115	11.8%
Middle-aged, methamphetamine use, current smoking	40	No	Yes	No	No	120	15.2%
Middle-aged, cocaine only, Black, current smoking	50	Yes	Yes	Yes	No	125	32.8%
Older, cocaine only, CV medications	60	No	No	Yes	Yes	135	55.0%
Older, multiple risk factors	70	Yes	Yes	Yes	Yes	145	74.7%

**Note**. Predicted 3-year CVD risk estimated using the reduced model: P = 1/(1 + e^−η^), where η = −4.751 + 0.054(age) + 0.132(Black race) + 0.150(current cigarette smoking) + 0.301(cocaine use only) + 0.599(CV medications) + 0.006(systolic blood pressure). Binary predictors coded 1 if present, 0 otherwise; age in years; systolic blood pressure (SBP) in mmHg. CV meds = any cardiovascular medication use. Cocaine only = cocaine without methamphetamine use; reference category is methamphetamine only or concurrent cocaine and methamphetamine use.

### 3.7 Subgroup performance

#### 3.7.1 Subgroup performance by race and sex

We assessed subgroup performance by race and sex as a secondary fairness analysis (Table S3). Mean calibration was excellent across all subgroups: O/E ratios were 1.00 and calibration-in-the-large was near zero for Black patients, White patients, female patients, and male patients alike, indicating no systematic over- or underprediction in any group. Discrimination was modestly lower in White patients (C-statistic 0.689, 95% CI 0.662–0.716) than in Black patients (0.712, 0.696–0.728) or male patients (0.734, 0.717–0.750), though confidence intervals overlapped substantially. The ICI was somewhat higher in Black patients (0.015) than in other subgroups (0.008–0.012), suggesting greater mean absolute calibration error within this group despite accurate mean prediction; this likely reflects heterogeneity in risk factor distributions within a large and clinically diverse subgroup. Calibration slopes were within acceptable range across all groups, with no subgroup showing evidence of meaningful systematic miscalibration. These findings support the equitable performance of the model across the race and sex subgroups with sufficient sample sizes for evaluation.

#### 3.7.2 Subgroup performance by stimulant type

The subgroup results support the pooled model approach (Table S4). C-statistics are consistent across cocaine-only (0.72) and methamphetamine-only (0.71), with slightly lower and less precise discrimination in poly-stimulant (0.69) as expected given n = 261. Calibration is excellent for cocaine-only and methamphetamine-only, with O/E near 1, CITL near 0, slopes near 1. The poly-stimulant group shows meaningful miscalibration signals (O/E = 1.21, CITL = 0.25), suggesting the pooled model systematically underpredicts risk in that group, but the CIs are very wide given the small n and none formally exclude the null. The ICI is notably higher for poly-stimulant (0.036 vs 0.011/0.009), consistent with worse local calibration, but again driven largely by sample size.

## 4 Discussion

This 3-year cardiovascular risk prediction model for adults who use stimulants demonstrated excellent calibration and clinically meaningful risk stratification in a large EHR-based cohort. The model meaningfully extends existing risk prediction tools by focusing on a shorter time horizon and incorporating stimulant-related characteristics in a cohort with substantial cardiovascular comorbidity. As a first-generation model developed to establish proof of concept, its primary contribution is demonstrating that meaningful cardiovascular risk stratification is achievable in this population using routinely available EHR data; subsequent iterations incorporating richer longitudinal and biomarker data are expected to improve discrimination. Notably, a six-predictor reduced model achieved identical discrimination and comparable calibration, suggesting the core predictive signal is robust and implementable without laboratory data.

### 4.1 Principal findings

In a cohort of 6940 adults with documented cocaine or methamphetamine use who were followed for at least 3 years, nearly 30% experienced a major cardiovascular event within 3 years, underscoring the substantial short-term risk faced by this population. The final LASSO-selected model retained eight predictors spanning traditional risk factors, stimulant exposure, race, and cardiovascular medication use, and achieved a C-statistic of 0.73 with excellent agreement between mean predicted and observed risk and close alignment across deciles of predicted risk. These findings indicate the model provides well-calibrated absolute risk estimates across the full spectrum of predicted risk.

Higher 3-year cardiovascular risk was associated with older age, current cigarette smoking, higher systolic blood pressure, and cocaine use (versus methamphetamine use or use of both substances), consistent with the known contributions of traditional cardiovascular risk factors and stimulant exposure to myocardial infarction, stroke, and heart failure. The association between cardiovascular medication use and increased risk most likely reflects confounding by indication: patients prescribed antihypertensive agents or statins carry clinically recognized cardiovascular risk regardless of whether those medications were initiated for primary prevention or established disease. In prediction models, medication variables commonly carry positive coefficients despite beneficial treatment effects precisely because they mark underlying disease burden; the AHA PREVENT equations take the same approach, incorporating positive coefficients for antihypertensive use. The model’s ability to recapitulate expected directions of association for key predictors, while also highlighting stimulant-related variables, supports both its face validity and its grounding in established cardiovascular mechanisms.

### 4.2 Relation to prior work

Existing cardiovascular risk prediction models, including the AHA PREVENT equations, were derived primarily in general populations with relatively low prevalence of substance use disorders and are designed for 10- and 30-year horizons. In contrast, individuals who use stimulants experience cardiovascular events through a combination of accelerated atherosclerosis and acute toxic effects that may occur over much shorter time frames and at younger ages than those captured by traditional models. Prior studies of nonmedical stimulants have documented early increases in myocardial infarction, stroke, arrhythmias, and heart failure, particularly in the weeks to months following exposure, as well as long-term structural cardiac changes such as cardiomyopathy (Borelli and Cheng, 2026, Leyde et al., 2025, Tobolski et al., 2022, Duflou, 2020, O'Keefe et al., 2022). A 3-year horizon therefore is better suited to the clinically relevant decision window for patients who use cocaine or methamphetamine and for clinicians weighing preventive interventions in the context of ongoing stimulant exposure.

### 4.3 Missing data

Missing data present well-recognized challenges in EHR-based prediction model development, validation, and implementation (Lin et al., 2025). In this cohort, lipid panels, hemoglobin A1c, and eGFR were missing at rates that would have substantially reduced the analytic sample had they been required predictors; their exclusion was therefore prespecified on both analytic and clinical grounds. The missingness of laboratory values in this population is likely not missing completely at random: people who use stimulants frequently have fragmented care, and the absence of a lipid panel or hemoglobin A1c may itself be informative, reflecting disengagement from preventive care rather than low clinical risk. Requiring these variables would have conditioned model inclusion on laboratory availability, introducing selection bias and limiting transportability to the settings most likely to serve this population. The model was therefore designed around predictors expected to be consistently available at the point-of-care across diverse clinical settings, including primary care, emergency departments, and addiction treatment programs. Informative missingness is not unique to stimulant-using populations: in the AHA PREVENT derivation cohorts, hemoglobin A1c, an optional predictor, was missing at very high rates, suggesting that missingness among core required predictors such as lipids and diabetes status was likely substantial as well, though these rates were not reported (Khan et al., 2024, Hong et al., 2026).

### 4.4 Clinical implications

These findings have implications for clinical practice. The high 3-year event rate observed in this cohort indicates that adults who use stimulants constitute a population in whom aggressive cardiovascular risk assessment and prevention are warranted. A calibrated 3-year risk prediction tool can support shared decision-making about statin initiation, intensity of blood pressure control, and deployment of cardioprotective therapies in settings where patients may have unstable housing, intermittent engagement with care, or competing acute needs. For example, a patient with cocaine use, current cigarette smoking, and elevated systolic blood pressure whose 3-year risk is estimated at 30–35% might reasonably be prioritized for immediate initiation or intensification of risk-reducing therapies, coupled with interventions to support stimulant cessation. Patients estimated to be at high short-term risk may also warrant referral for further cardiovascular evaluation, including assessment for structural heart disease or subclinical ischemia, given the well-described associations between stimulant use, particularly cocaine, and accelerated coronary artery disease and left ventricular dysfunction.

A caveat is in order regarding younger patients (those in their 20 s). Age in the model likely captures both cumulative stimulant exposure and age-related cardiovascular risk progression independent of substance use, and without structured data on duration of use these contributions cannot be disentangled. This limitation is most consequential at the lower end of the age range, where a 21-year-old with several years of heavy use and one with six months of use receive the same age-based contribution to predicted risk despite meaningfully different clinical profiles. Predictions in this age group should therefore be interpreted with caution pending external validation in cohorts with richer exposure data.

The short-term risk horizon also reflects the realities of care for many individuals who use stimulants, who may not reliably engage with longitudinal primary care or remain in the same health system for a decade. A 3-year prediction window is more actionable in emergency departments, inpatient units, and addiction treatment programs, where clinicians must make time-sensitive decisions about cardiovascular evaluation and secondary prevention. By quantifying near-term risk, the model may help frame conversations about the cardiovascular consequences of ongoing stimulant use in concrete, patient-centered terms, potentially enhancing motivation for behavior change more effectively than abstract lifetime risk estimates.

The inclusion of stimulant-related variables also underscores the importance of systematically documenting substance use in the EHR and integrating addiction and cardiovascular care. The independent association of cocaine use with risk suggests that stimulant type is not merely a proxy for broader social or behavioral risk but may capture distinct pathophysiological pathways. This finding supports efforts to develop care models in which cardiology, primary care, and addiction medicine collaborate to identify and manage high-risk stimulant-using patients.

### 4.5 Inclusion of race in the prediction model

The full model includes Black race as a predictor, departing from the race-free design of the AHA PREVENT equations. PREVENT excluded race on the grounds that its inclusion might be misinterpreted as implying genetic or biological determinism, offering the zip code-based Social Deprivation Index (SDI) as an optional alternative (Khan et al., 2023, Khan et al., 2024, Khan et al., 2026). However, area-level proxies are subject to ecological fallacy, a liability for individualized risk prediction. In our cohort, Black patients experienced a 3-year event rate of 35.9% compared with 20.7% in White patients; Black race was selected by LASSO and contributed independently to predicted risk after adjustment for all other model predictors. Based on the reduced model coefficient for Black race (β = 0.11, OR 1.12), removing race from the model would underestimate predicted risk by approximately 3 percentage points for a Black patient at mean predicted risk, with larger absolute underestimates at higher risk levels. Given that Black patients in this cohort had a mean event rate nearly 15 percentage points higher than White patients, this bias would fall most heavily on the group with the greatest cardiovascular burden.

### 4.6 Limitations and future directions

Several limitations warrant consideration. First, the study relies on EHR data from a single academic health system in the mid-Atlantic region, which may limit generalizability to other populations. Before embedding the model into clinical workflows, external validation in diverse settings is needed to assess its performance; this includes validation in western U.S. settings where methamphetamine use is more prevalent (Substance Abuse and Mental Health Services Administration SAMHSA, 2025).

Second, the model was not adjusted for competing risks from non-cardiovascular mortality, as mortality data were unavailable in our dataset. While ignoring competing risks can theoretically overestimate absolute cardiovascular risk, this bias is most consequential over longer horizons and in older adults (Hageman et al., 2023, Cooper et al., 2022), and is substantially mitigated here by the 3-year prediction horizon. The apparent excess all-cause mortality observed among people who use stimulants likely reflects, at least in part, confounding by social determinants of health rather than stimulant-attributable competing mortality per se (Kariisa et al., 2022, Blair and Siddiqi, 2022, Monnat, 2018).

Third, the derivation cohort is conditioned on health system engagement prior to a cardiovascular event; patients whose first documented contact was precipitated by a cardiovascular event are excluded. While this reduces the likelihood that early symptoms prompted the encounter at which stimulant use was first documented, the index encounter may nonetheless reflect years of prior unrecognized exposure. The model is therefore intended for clinically engaged patients with recognized stimulant use, not for those presenting for the first time with an acute event.

Further, the model estimates conditional 3-year cardiovascular risk among clinically engaged patients, the relevant estimand for point-of-care decision support where accurate near-term absolute risk is needed. The intended clinical application is risk communication at the point-of-care, where any modest upward bias in absolute risk estimates, if present, would err toward rather than away from preventive intervention, a conservative direction given the underrecognition of cardiovascular risk in this population. In future external validation studies with complete capture of non-cardiovascular mortality, we plan to compare our logistic model's 3-year risk estimates with those from competing-risk approaches (e.g., Fine-Gray) to quantify and, if necessary, correct any residual overestimation.

The model also lacks granular information about stimulant exposure that may be important determinants of cardiovascular risk. Duration of use, route of administration, and severity of stimulant use disorder were not available in structured EHR data and could not be included as predictors. Route of administration is particularly relevant given evidence that smoked and intravenous cocaine and methamphetamine may confer greater cardiovascular toxicity than intranasal use through more rapid drug delivery and higher peak plasma concentrations. Polysubstance use beyond the cocaine-methamphetamine distinction captured here – including concurrent alcohol, opioid, or cannabis use – was similarly unavailable as a structured predictor, despite evidence that cocaethylene formation from concurrent cocaine and alcohol use confers additional cardiotoxicity beyond either substance alone. To the extent that these unmeasured exposure characteristics are associated with both stimulant use severity and cardiovascular risk, their omission may attenuate the model's discrimination. That the model achieves a C-statistic of 0.73 using only routinely documented EHR variables suggests meaningful signal is recoverable without these data; incorporating richer exposure information in future iterations, potentially through structured substance use assessments integrated into the EHR, represents a clear path toward improved individual-level risk discrimination.

Finally, the present study does not include a formal clinical impact evaluation. The evidence base for cardiovascular prevention among people who use stimulants is actively evolving but remains largely extrapolated from general population data. Preclinical studies suggest potential cardiovascular benefits of statins in cocaine and methamphetamine exposure models, though to our knowledge no randomized human trials have evaluated statins for chronic cardiovascular risk reduction in stimulant-using populations (Sáez et al., 2014, Coffin and Suen, 2023). Clinicians using this tool to guide statin initiation should be aware that the model may underestimate the full benefit of treatment, since potential neurological and other pleiotropic effects of statins would not be reflected in predicted cardiovascular risk estimates alone. The recent American Society of Addiction Medicine/American Academy of Addiction Psychiatry clinical practice guideline for stimulant use disorder recommends heightened clinical suspicion for cardiac disorders but does not address long-term cardiovascular risk reduction strategies (Clinical Guideline Committee CGC Members et al., 2024). Our model is intended to help address this gap by providing individualized risk estimates that may support preventive decision-making. Prospective implementation studies evaluating effects on clinician behavior and patient outcomes are needed.

## 5 Conclusions

Among adults with documented cocaine or methamphetamine use, a 3-year cardiovascular risk prediction model grounded in EHR data and developed with modern penalized regression produced absolute risk estimates that closely match observed outcomes across the full spectrum of predicted risk. These findings are best understood as establishing proof of concept and providing a validated foundation for the next phase of this work, which includes external validation in independent cohorts, assessment of calibration transportability, and implementation studies examining whether risk communication informed by stimulant-specific data influences clinical decision-making and supports cardiovascular disease prevention in this population.

## CRediT authorship contribution statement

**Rebecca Arden Harris:** Writing – review & editing, Writing – original draft, Visualization, Validation, Software, Methodology, Formal analysis, Data curation, Conceptualization. **Fengge Wang:** Writing – review & editing, Writing – original draft, Methodology. **Warren B. Bilker:** Writing – review & editing, Methodology. **Renae Judy:** Writing – review & editing, Writing – original draft, Methodology. **Michael G. Levin:** Writing – review & editing, Writing – original draft, Methodology. **Scott M. Damrauer:** Writing – review & editing, Writing – original draft, Methodology. **Sean Hennessy:** Writing – review & editing, Writing – original draft, Supervision, Methodology, Formal analysis, Conceptualization.

## Funding/support

Dr. Harris’ research is supported by the 10.13039/100000026National Institute on Drug Abuse (K23 DA054157).

## Declaration of Competing Interest

none

## Footnotes

Appendix A Supplementary data associated with this article can be found in the online version at doi:10.1016/j.dadr.2026.100459.
