# Supplementary material for: Three-year cardiovascular risk prediction among people who use cocaine or methamphetamine
Source: Drug Alcohol Depend Rep. 2026 Jun 22;20:100459. doi: 10.1016/j.dadr.2026.100459 (PMC13380514; doi:10.1016/j.dadr.2026.100459)
Supplement: Supplementary file 1 — Supplementary material [file mmc1.docx]

Three-year cardiovascular risk prediction among people who use cocaine or methamphetamine

# Rebecca Arden Harris, MD, MSc

# Fengge Wang, PhD

# Warren B. Bilker, PhD, MS

# Renae Judy, MS

# Michael G. Levin, MD

# Scott M. Damrauer, MD

# Sean Hennessy, PharmD, PhD

**Supplemental Material**

**Table S1.** Comparison of ICD-10 outcome codes: Stimulant-CVD study vs. AHA PREVENT

**Table S2.** Threshold-dependent performance metrics for the reduced 6-predictor model

**Table S3.** Subgroup performance by race and sex

**Table S4.** Subgroup performance by stimulant type

**Table S5. Component-level model performance, optimism-corrected**

| **Table S1.** Comparison of ICD-10 outcome codes: Stimulant-CVD study vs. AHA PREVENT* | | | | | |
| --- | --- | --- | --- | --- | --- |
| **Outcome Category** | **Stim-CVD Study:**  **ICD-10 Codes** | **ICD-10 Code Descriptions** | **PREVENT ICD-10 Codes** | **Additional Coverage of Stim-CVD study vs. PREVENT** | **Rationale for Expanded Codes**  **in Stimulant- CVD Study** |
| **Ischemic heart disease** | I20.xx  to  I25.xx | Unstable angina (I20); acute MI (I21); subsequent MI (I22); post-MI complications (I23); other acute IHD (I24); chronic IHD (I25) | I21.xx  I22.xx | I20 (unstable angina); I23 (post-MI complications); I24 (other acute IHD); I25 (chronic IHD) | Cocaine and methamphetamine cause coronary vasospasm and accelerate atherosclerosis, frequently presenting as unstable angina or non‑MI ischemia (I20, I24) rather than completed infarction (Slezak et al., 2025; Saunders-Hastings et al., 2021). Limiting surveillance to I21–I22 would miss clinically important events; chronic IHD (I25) captures the accumulated ischemic burden in younger, long‑term users. Per CDC indicator definitions (CDC, 2024), we use the extended ICD‑10 code set (I20–I25) to capture ischemic presentations associated with stimulant use. |
| **Cerebrovascular disease** | I60.xx  to  I69.xx | Subarachnoid hemorrhage (I60); intracerebral hemorrhage (I61); other intracranial hemorrhage (I62); cerebral infarction (I63); stroke NOS (I64); cerebral artery occlusion without infarction (I65-I66); other cerebrovascular disease (I67); cerebrovascular disorders in diseases elsewhere (I68); sequelae (I69) | I61.xx  I62.xx  I63.xx | I60 (subarachnoid hemorrhage); I64 (stroke NOS); I65-I66 (cerebral artery occlusion without infarction); I67-I69 (other cerebrovascular disease and sequelae) | Surveillance limited to I60–I63 would miss unspecified stroke (I64), later effects (I69), and related vascular codes such as occlusion/stenosis of precerebral and cerebral arteries (I65–I66) and other cerebrovascular diseases (I67); I68 can be included when cerebrovascular disorders are coded in association with other diseases (Columbo et al., 2024). These omissions would undercount stimulant‑related cerebrovascular burden. Per CDC indicator definitions (CDC, 2024), we include I60–I69 to capture acute, unspecified, related‑vascular, and chronic stroke presentations. |
| **Heart failure** | I50.xx | Heart failure, all subtypes (systolic, diastolic, combined, unspecified) | I50.xx | *None (identical to PREVENT)* | Stimulant-associated cardiomyopathy, particularly methamphetamine-related dilated cardiomyopathy, manifests clinically as heart failure and captured under I50 (Pocobelli et al., 2022; Bosco-Lévy et al., 2019; Bates et al., 2023; Goyal et al., 2020; Klein et al., 2025). No expansion beyond PREVENT’s definition was required. Although cardiomyopathy (I42) was not separately included in the primary composite – consistent with PREVENT – cardiomyopathy and heart failure were nearly perfectly correlated in our health system-wide diagnosis counts for persons using stimulants across the study period (Pearson R^2^ = 0.99), with a regression intercept indistinguishable from zero (p = 0.825), indicating that cardiomyopathy events scaled proportionately with heart failure diagnoses across the full range of observation years. These findings indicate that the large majority of clinically significant cardiomyopathy events are captured through I50, and the omission of I42 as a standalone code introduces negligible outcome misclassification. |

Abbreviations: AHA = American Heart Association; ASCVD = atherosclerotic cardiovascular disease; EHR = electronic health record; ICD-10 = International Classification of Diseases, Tenth Revision; IHD = ischemic heart disease; MI = myocardial infarction; NOS = not otherwise specified; HF = heart failure; PREVENT = Predicting Risk of Cardiovascular Disease EVENTs. PREVENT ICD-10 codes are as reported in Khan et al. (2024, Supplement Appendix 1, Table 1.6).

Note ICD‑10 codes use a prefix and optional subcode (digits left and right of the decimal); when a code is shown with no digits to the right of the decimal it means the search included that prefix and all its more specific subcodes (e.g., I21.xx includes I21.0, I21.1). Component-level model performance is reported in Table S5.

*Cardiac arrhythmias (I44–I49) were examined as an exploratory endpoint given strong biological plausibility (cocaine blocks cardiac sodium channels predisposing to ventricular arrhythmias; methamphetamine promotes structural remodeling that creates a chronic arrhythmogenic substrate) but were excluded from the primary composite for two reasons. First, no major cardiovascular risk prediction framework, including PREVENT, includes arrhythmias, reflecting consensus that arrhythmic events arise through mechanisms distinct from the atherosclerotic pathways these models target. Second, the I44–I49 range is diagnostically heterogeneous in EHR data, spanning benign incidental findings to life-threatening events, risking dilution of harder endpoints. Consistent with these concerns, the arrhythmia model showed markedly lower discrimination than the primary components (optimism-corrected C-statistic 0.579) despite excellent calibration, a pattern more consistent with episodic, trigger-dependent events than with the chronic risk factor burden the model captures. Defining a stimulant-specific arrhythmia sub-phenotype is a priority for future work.

**References**

Bates BA, Akhabue E, Nahass MM, et al. Validity of International Classification of Diseases (ICD)-10 diagnosis codes for identification of acute heart failure hospitalization and heart failure with reduced versus preserved ejection fraction in a national Medicare sample. Circ Cardiovasc Qual Outcomes. 2023;16(2):e009078.

Bosco-Lévy P, Duret S, Picard F, et al. Diagnostic accuracy of the International Classification of Diseases, Tenth Revision, codes of heart failure in an administrative database. Pharmacoepidemiol Drug Saf. 2019;28(2):194-200.

Columbo JA, Daya N, Colantonio LD, et al. Derivation and validation of ICD-10 codes for identifying incident stroke. JAMA Neurol. 2024;81(8):875-881.

Goyal P, Bose B, Creber RM, et al. Performance of electronic health record diagnosis codes for ambulatory heart failure encounters. J Card Fail. 2020;26(12):1060-1066.

Klein S, Mukhopadhyay A, Hamo CE, et al. Accuracy of electronic health record-based definitions for patients with heart failure. Am J Med. 2025;S0002-9343(25)00435-8.

Pocobelli G, Ichikawa L, Yu O, et al. Validation of International Classification of Diseases, Tenth Revision, Clinical Modification diagnosis codes for heart failure subtypes. Pharmacoepidemiol Drug Saf. 2022;31(9):992-997.

Saunders-Hastings P, Heong SW, Srichaikul J, et al. Acute myocardial infarction: Development and application of an ICD-10-CM-based algorithm. PLoS One. 2021;16(7):e0253580. doi:https://doi.org/10.1371/journal.pone.0253580

Slezak J, Bruxvoort KJ, Sy LS, et al. Validation of ICD-10 diagnosis codes for identification of acute myocardial infarction. Pharmacoepidemiol Drug Saf. 2025;34(7):e70179. doi:https://doi.org/10.1002/pds.70179

**Table S2.** Threshold-dependent performance metrics for the reduced 6-predictor model

| **Metric** | **Threshold: 20%** | **Threshold: 30%** | **Threshold: 40%** |
| --- | --- | --- | --- |
| Sensitivity | 84.9% | 70.0% | 50.8% |
| Specificity | 45.5% | 64.5% | 80.3% |
| Positive predictive value (PPV) | 40.1% | 45.8% | 52.6% |
| Negative predictive value (NPV) | 87.5% | 83.3% | 79.2% |
| Classified higher-risk, % | 63.6% | 45.9% | 29.0% |
| Classified lower-risk, % | 36.4% | 54.1% | 71.0% |

Abbreviations: PPV, positive predictive value; NPV, negative predictive value. Metrics are derived from the reduced 6-predictor model applied to the full analytic cohort. The 30% threshold corresponds approximately to the observed 3-year event rate (29.6%) and falls within the range at which the model demonstrated net benefit over treat-all and treat-none strategies in the decision curve analysis. The 20% and 40% thresholds are shown to illustrate how sensitivity–specificity tradeoffs shift across the clinically plausible decision range. These metrics supplement the primary calibration and discrimination analyses; their interpretation depends on clinical context and the relative costs of false-positive and false-negative classifications

These metrics illustrate the sensitivity-specificity tradeoff inherent in any threshold-based classification. At the 40% threshold, the model is most selective: 29.0% of patients are classified as higher-risk, specificity is 80.3%, and PPV is 52.6%, meaning that among those flagged as higher-risk, approximately half will experience a cardiovascular event within 3 years. As the threshold is lowered to 30%, which corresponds approximately to the observed event rate, more patients are classified as higher-risk (45.9%), sensitivity increases to 70.0%, and specificity remains reasonable at 64.5%. Lowering the threshold further to 20% captures the greatest proportion of patients who will experience an event (sensitivity 84.9%), though at the cost of classifying nearly two-thirds of the cohort (63.6%) as higher-risk. The NPV of 83.3% at the 30% threshold indicates that among patients classified as lower-risk, approximately five in six will remain event-free over 3 years, a finding that may inform deferral of intensive preventive intervention in that group, pending clinical judgment. Across all thresholds, PPV remains modest (40–53%), reflecting the inherent difficulty of predicting individual events in a population where baseline risk is already elevated. These characteristics are consistent with a model designed for absolute risk estimation rather than binary screening, and should be interpreted alongside the calibration and discrimination metrics presented in the main text.

**Table S3.** Subgroup performance by race and sex

| **Metric** | **Black**  (n=4,109) | **White** (n=2,443) | **Female** (n=2,844) | **Male**  (n=4,096) |
| --- | --- | --- | --- | --- |
| Event rate | 35.9% | 20.7% | 29.0% | 30.8% |
| C-statistic | 0.712 | 0.689 | 0.723 | 0.734 |
| 95% CI | 0.696–0.728 | 0.662–0.716 | 0.702–0.744 | 0.717–0.750 |
| O/E ratio | 1.000 | 1.000 | 1.000 | 1.000 |
| 95% CI | 0.959–1.041 | 0.921–1.080 | 0.942–1.058 | 0.953–1.047 |
| Calibration-in-the-large (CITL) | 0.000 | 0.001 | 0.000 | 0.000 |
| 95% CI | −0.068–0.068 | −0.105–0.106 | −0.089–0.089 | −0.073–0.073 |
| Calibration slope | 1.045 | 0.923 | 0.959 | 1.030 |
| 95% CI | 0.948–1.142 | 0.788–1.058 | 0.851–1.067 | 0.937–1.123 |
| Integrated Calibration Index (ICI) | 0.015 | 0.012 | 0.012 | 0.008 |
| 95% CI | 0.008–0.029 | 0.006–0.030 | 0.006–0.028 | 0.005–0.023 |
| Brier score | 0.202 | 0.150 | 0.180 | 0.183 |
| 95% CI | 0.197–0.207 | 0.140–0.159 | 0.172–0.187 | 0.177–0.189 |

Abbreviations: CI, confidence interval; O/E, observed/expected; ICI, integrated calibration index. Performance metrics are based on apparent model predictions from the full 8-predictor model applied to the full analytic cohort. The C-statistic 95% CI is asymptotic normal; CITL and calibration slope 95% CIs are analytic; O/E ratios and Brier score 95% CI use a delta-method standard error. ICI 95% CIs are bootstrap percentile (500 replicates per subgroup).

**Table S4.** Subgroup performance by stimulant type

| **Metric** | **Cocaine-only**  (n=5,604) | **Methamphetamine-only**  (n=1,075) | **Poly-stimulant use**  (n=261) |
| --- | --- | --- | --- |
| Event rate | 32.5% | 16.5% | 21.1% |
| C-statistic | 0.717 | 0.710 | 0.690 |
| 95% CI | 0.702–0.731 | 0.667–0.754 | 0.605–0.775 |
| O/E ratio | 1.000 | 0.951 | 1.206 |
| 95% CI | 0.962–1.038 | 0.820–1.082 | 0.911–1.500 |
| Calibration-in-the-large (CITL) | -0.000 | -0.068 | 0.254 |
| 95% CI | −0.061–0.061 | −0.242–0.107 | −0.070–0.578 |
| Calibration slope | 1.008 | 0.953 | 1.014 |
| 95% CI | 0.926–1.089 | 0.744–1.162 | 0.572–1.456 |
| Integrated Calibration Index (ICI) | 0.011 | 0.009 | 0.036 |
| 95% CI | 0.005–0.022 | 0.007–0.034 | 0.016–0.089 |
| Brier score | 0.194 | 0.126 | 0.150 |
| 95% CI | 0.188–0.198 | 0.112–0.140 | 0.118–0.181 |

Abbreviations: CI, confidence interval; O/E, observed/expected; ICI, integrated calibration index. Performance metrics are based on apparent model predictions from the full 8-predictor model applied to the full analytic cohort. The C-statistic 95% CI is asymptotic normal; CITL and calibration slope 95% CIs are analytic; O/E ratios and Brier score 95% CI use a delta-method standard error. ICI 95% CIs are bootstrap percentile (500 replicates per subgroup).

**Table S5. Component-level model performance, optimism-corrected**

| **Outcome**  **Components** | **Event**  **rate** | **C-statistic**  **(95% CI)** | **O/E Ratio**  **(95% CI)** | **ICI**  **(95% CI)** |
| --- | --- | --- | --- | --- |
| CBV | 15.5% | 0.713 (0.696–0.729) | 0.977 (0.925–1.032) | 0.008 (0.001–0.015) |
| HF | 12.0% | 0.700 (0.680–0.719) | 0.991 (0.930–1.055) | 0.004 (-0.002–0.010) |
| ISH | 16.5% | 0.716 (0.701–0.732) | 0.986 (0.939–1.041) | 0.011 (0.003–0.018) |
| Composite | 29.6% | 0.728 (0.713–0.741) | 0.988 (0.952–1.022) | 0.010 (0.001–0.018) |
| ARR | 32.3% | 0.579 (0.565–0.594) | 0.994 (0.961–1.028) | 0.010 (0.001–0.020) |

**Abbreviations: CBV, cerebrovascular disease; HF, heart failure; ICI, integrated calibration index; ISH, ischemic heart disease; ARR, Arrhythmia; O/E, observed/expected ratio. See Table S1 for ICD‑10 definitions of component outcomes.**

To evaluate whether the composite outcome model performed consistently across its constituent components, we assessed discrimination and calibration separately for ischemic heart disease, cerebrovascular disease, and heart failure. These analyses used the full 8-predictor model with optimism-corrected performance estimates derived from 500 bootstrap replicates. Component-level outcomes were defined independently: each patient was classified as an event for a given component if that diagnosis occurred during the 3-year follow-up window, regardless of whether another component event occurred first. Patients may therefore contribute to more than one component row; component event counts are not mutually exclusive and sum to more than the composite event rate of 29.6%.

Discrimination was remarkably consistent across all three components. C-statistics were 0.716 (95% CI 0.701–0.732) for ischemic heart disease, 0.713 (95% CI 0.696–0.729) for cerebrovascular disease, and 0.700 (95% CI 0.680–0.719) for heart failure — a narrow range spanning only 0.016 — and all three were comparable to the composite C-statistic of 0.728 (95% CI 0.713–0.741). This consistency indicates that composite model performance is not driven by any single component and that the predictors generalize across the distinct pathophysiological mechanisms underlying each outcome. Calibration was excellent across all three components, with O/E ratios ranging from 0.977 to 0.991 and ICI values of 0.008–0.011, closely mirroring the composite calibration metrics and indicating no systematic over- or underprediction for any individual component.

Arrhythmia is reported separately in Table S5 given its distinct status as an exploratory outcome outside the PREVENT framework. Despite having the highest event rate in the cohort (32.3%), the arrhythmia model showed markedly lower discrimination than the three primary components, with an optimism-corrected C-statistic of 0.579 (95% CI 0.565–0.594). Minimal bootstrap optimism (0.005) confirms that this reflects a genuine signal rather than overfitting. Calibration, however, was excellent: the O/E ratio was 0.994 and ICI 0.010, indicating accurate estimation of average arrhythmia risk despite poor individual-level discrimination. The divergence between calibration and discrimination is informative. The standard cardiovascular risk factors included in the model – demographics, cigarette smoking, cocaine-only use, systolic blood pressure, and cardiovascular medication use – appear sufficient to estimate the *population-level* burden of stimulant-associated arrhythmia but cannot reliably distinguish which individuals will be affected. This may reflect the underlying pathophysiology: stimulant-associated arrhythmia is more episodic and trigger-dependent than myocardial infarction or heart failure, and less tied to the chronic risk factor burden that the model captures. Improving individual-level arrhythmia discrimination in this population would likely require predictors not available in routine EHR data, such as QTc interval, electrolyte abnormalities, prior arrhythmia history, and granular details about stimulant use frequency and pattern. These findings establish a performance benchmark and identify specific data requirements for a future arrhythmia-specific prediction tool.
